# Low latency global carbon budget indicates reduced land carbon sink in the year 2024

**DOI:** 10.1093/nsr/nwaf594

**Published:** 2026-01-15

**Authors:** Philippe Ciais, Piyu Ke, Yitong Yao, Stephen Sitch, Wei Li, Yidi Xu, Xiaomeng Du, Xiaofan Gui, Ana Bastos, Sönke Zaehle, Ben Poulter, Thomas Colligan, Auke M van der Woude, Wouter Peters, Zhu Liu, Zhe Jin, Xiangjun Tian, Yilong Wang, Junjie Liu, Sudhanshu Pandey, Chris O’Dell, Jiang Bian, Chuanlong Zhou, John Miller, Xin Lan, Jefferson Goncalves De Souza, Michael O’Sullivan, Pierre Friedlingstein, Guido R van der Werf, Glen P Peters, Frédéric Chevallier

**Affiliations:** Laboratoire des Sciences du Climat et de l’Environnement, University Paris Saclay CEA CNRS, France; Laboratoire des Sciences du Climat et de l’Environnement, University Paris Saclay CEA CNRS, France; Department of Earth System Science, Tsinghua University, China; Faculty of Environment, Science and Economy, University of Exeter, UK; Department of Earth and Environmental Engineering, Columbia University, USA; Faculty of Environment, Science and Economy, University of Exeter, UK; Department of Earth System Science, Tsinghua University, China; Laboratoire des Sciences du Climat et de l’Environnement, University Paris Saclay CEA CNRS, France; Department of Earth System Science, Tsinghua University, China; Machine learning group, Microsoft research, China; Institute for Earth System Science and Remote Sensing, Leipzig University, Germany; Biogeochemical Signals Department, Max Planck Institute for Biogeochemistry, Germany; Spark Climate Solutions, USA; Earth System Science Interdisciplinary Center, University of Maryland, College Park, USA; Environmental Sciences Group, Dept of Meteorology and Air Quality, Wageningen University, the Netherlands; Environmental Sciences Group, Dept of Meteorology and Air Quality, Wageningen University, the Netherlands; Department of Earth System Science, Tsinghua University, China; Institute of Carbon Neutrality, Sino-French Institute for Earth System Science, College of Urban and Environmental Sciences, Peking University, China; State Key Laboratory of Tibetan Plateau Earth System, Environment and Resources (TPESER), Institute of Tibetan Plateau Research, Chinese Academy of Sciences, China; State Key Laboratory of Tibetan Plateau Earth System, Environment and Resources (TPESER), Institute of Tibetan Plateau Research, Chinese Academy of Sciences, China; Jet Propulsion Laboratory, California Institute of Technology, Pasadena, USA; Jet Propulsion Laboratory, California Institute of Technology, Pasadena, USA; Cooperative Institute for Research in the Atmosphere, Colorado State University, Fort Collins, USA; Machine learning group, Microsoft research, China; Laboratoire des Sciences du Climat et de l’Environnement, University Paris Saclay CEA CNRS, France; National Oceanic and Atmospheric Administration Global Monitoring Laboratory, USA; National Oceanic and Atmospheric Administration Global Monitoring Laboratory, USA; Cooperative Institute for Research in Environmental Sciences, University of Colorado Boulder, USA; Faculty of Environment, Science and Economy, University of Exeter, UK; Faculty of Environment, Science and Economy, University of Exeter, UK; Faculty of Environment, Science and Economy, University of Exeter, UK; Laboratoire de Météorologie Dynamique, IPSL, CNRS, ENS, Université PSL, Sorbonne Université, École Polytechnique, France; Environmental Sciences Group, Dept of Meteorology and Air Quality, Wageningen University, the Netherlands; CICERO Center for International Climate Research, Norway; Laboratoire des Sciences du Climat et de l’Environnement, University Paris Saclay CEA CNRS, France

In 2024, the atmospheric CO_2_ growth rate based on the globally averaged marine boundary layer (MBL) observations from the National Oceanic and Atmospheric Administration (NOAA) network reached 3.73 ± 0.08 ppm yr^−1^, marking a record high since continuous measurements began in 1959 (Fig. 1a) [1]. The whole-atmosphere growth rate derived from independent OCO-2 satellite observations for 2024 was 3.20 ± 0.1 ppm yr^−1^ using the Growth Rates from Satellite Observations data-driven approach (GRESO) from Ref. [2], the highest value of the OCO-2 record since 2015. The whole-atmosphere growth rate derived from our flux inversion models assimilating OCO-2 observations mainly over land for 2024 was 3.23 ± 0.12 ppm yr^−1^, thus unsurprisingly being almost equal to GRESO and less than the MBL stations but still a record high in the OCO-2 inversions record since 2015. This 38.15% increase in the CO_2_ growth rate between 2023 and 2024 occurred despite fossil fuel CO_2_ emissions increasing by only 0.85% [3]. This highlights an unprecedented weakening of the net carbon uptake on land and ocean. Net land uptake is defined here as the sum of non-fossil land CO_2_ fluxes including photosynthesis, respirations, fire, rivers and land-use change emissions. Here, we present a low latency global and regional carbon budget for 2024, using top-down inversions and bottom-up models, revealing a strong weakening of the global net land carbon sink and widespread transitions of terrestrial regions from carbon sinks to sources.

**Figure 1.**
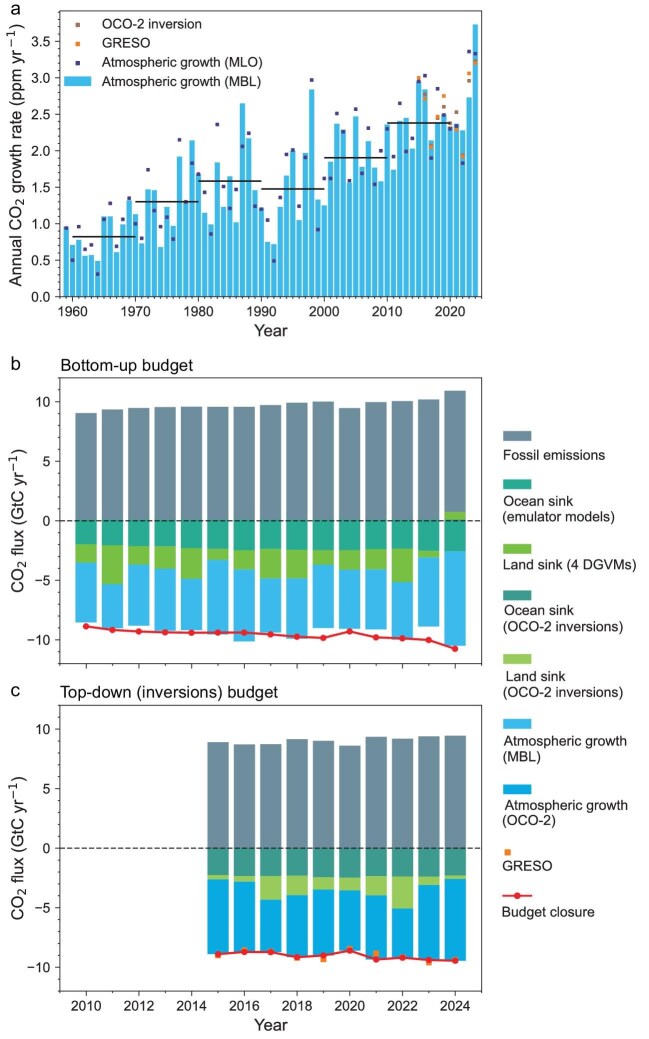
Atmospheric CO_2_ growth rate from 1959–2024 and global carbon budget from 2010–2024. (a) Annual June–July to June–July growth rate from marine boundary layer surface stations (MBL, blue bars), OCO-2 (dark brown squares) and the Growth Rates Using Satellite Observations approach from Ref. [2] (GRESO, orange squares). Our analysis is based on MBL. MBL is the average of many surface locations, which gives a better estimate of the global increase in atmospheric CO_2_ compared to the single high altitude MLO (Mauna Loa Observatory) estimate. (b) Global CO_2_ bottom-up budget obtained with our estimates of fossil CO_2_ emissions, DGVM-based net land CO_2_ flux, net ocean sink from ocean model emulators, and annual CO_2_ growth rates from MBL. The red curve is −1 × fossil emissions and the difference between the bars and this curve is the imbalance of the bottom-up budget. (c) Global CO_2_ top-down budget obtained with our estimates of fossil CO_2_ emissions and inversions assimilating OCO-2 atmospheric CO_2_ mixing ratios. By design, inversions match OCO-2 data and their growth rate is the blue bar. The orange squares are the CO_2_ growth rate obtained directly from OCO-2 data using the GRESO model.

We combined bottom-up estimates from four dynamic global vegetation models (DGVMs): JULES, OCN and ORCHIDEE-MICT and LPJ-EOSIM [4–6] and ocean biogeochemical model emulators [7] with top-down estimates from three atmospheric inversion models (CAMS, CMS-Flux and GONGGA) [8–10], which assimilate column CO_2_ concentration data from the OCO-2 satellite over land. The use of satellite data improves coverage over the tropics and continental areas, providing better spatial resolution and earlier detection of flux anomalies than *in situ* networks alone. The DGVMs

typically simulate fires with prognostic fire modules using population density, lightning ignitions, climate and simulated fuel moisture but have weaknesses in capturing extreme forest fires and tropical forest degradation fires. Therefore, we adjusted the DGVMs fluxes by subtracting their original fire emissions and adding the mean of the Global Fire Emissions Database (GFED4.1 s) [11] and the Global Fire Assimilation System (GFAS) [12] for the period 2010–2024. Our approach allows a rapid diagnosis of the most recent changes in the global carbon cycle and is consistent with protocols from the Global Carbon Budget (GCB) [3] although in this study we used the ERA5 climate forcing for DGVMs, instead of CRUJRA in the GCB protocol. In addition to the four low latency DGVMs listed above, we estimated the land CO_2_ flux using the SiB4 land flux model constrained for the global net CO_2_ flux by utilizing the Carbon Tracker Europe High-Resolution CTE-HR system [13] (0.1° × 0.1° globally) with a spatially upscaled version of the SiB4 land CO_2_ flux model (Fig. S1).

## THE GLOBAL CARBON BUDGET IN 2024

Figure 1b shows the bottom-up carbon budget, obtained by combining the average fossil emissions estimated by Carbon Monitor and previous projections of the Global Carbon Budget 2024 [3,14] with our bottom-up estimates of net land and ocean carbon fluxes. Figure 1c shows the top-down carbon budget based on the mean of the three OCO-2 inversions. Global fossil fuel and cement CO_2_ emissions were 10.19 ± 0.01 GtC yr^−1^ with a range of 10.18–10.19 GtC yr^−1^ from our two estimates. In the bottom-up budget, the DGVMs estimated a net land emission of 0.65 ± 0.85 GtC yr^−1^, continuing the downward trend observed in 2023, and becoming a net source—the weakest net land carbon flux since 1987, an estimate which includes fire and land use change emissions. This net land source contrasts with the decadal (2010–2022) average sink of 2.04 GtC yr^−1^. In the top-down budget for 2024, our inversions gave a small net land sink of 0.29 ± 0.80 GtC yr^−1^. The mean net land CO_2_ flux of the DGVMs and inversions was close to zero at 0.18 ± 0.58 GtC yr^−1^. The ocean CO_2_ uptake (without the river flux adjustment used in the GCB) for 2024 was 2.48 ± 0.29 GtC yr^−1^ (our ocean emulators: 2.66 ± 0.55 GtC yr^−1^, inversions: 2.30 ± 0.19 GtC yr^−1^) similar to the mean of the year 2023. The bottom-up budget imbalance, defined by the difference between fossil fuel emissions minus sinks from bottom-up models minus the observed CO_2_ growth rate, is 0.26 ± 1.03 GtC yr^−1^ using the growth rate of MBL stations and a conversion factor of 2.124 GtC per ppm. Such a positive imbalance indicates that our bottom-up models on average underestimate the global CO_2_ sink during that period. The inversions, by construction, match the observed CO_2_ growth rate of the OCO-2 satellite, which is less than the MBL by 1.06 GtC yr^−1^. The GFED4.1 s and GFAS fire emissions for 2024 are 2.35 GtC yr^−1^ and 1.79 GtC yr^−1^ compared to 2.34 GtC yr^−1^ and 1.97 GtC yr^−1^ in 2023 (Fig. S2). Relative to the 2015–2022 mean, global fire emissions in 2024 increased by 0.50 GtC yr^−1^, accounting for ∼20% of the 2024 net land sink reduction in our analysis.

## REGIONAL ANOMALIES

To gain insights into which regions caused the large reduction in the land sink in 2024, we analyzed quarterly spatial patterns of flux anomalies from the bottom-up models and OCO-2 inversions, using 2015–2022 as a reference period which corresponds to the period covered by the inversions. The ocean sink remained largely unchanged during this period. The results are displayed in Figs S3 and S1.

Over the ocean, the model emulators indicate a slight increase in carbon uptake, whereas the inversions suggest a slight decrease (Fig. S3). The carbon sinks in the Arctic Ocean, the Atlantic Ocean, the Indian Ocean, the Southern Ocean and coastal oceans based on emulators and inversions remained relatively unchanged. There is a divergence in the Pacific Ocean, where ocean model emulators suggest an unchanged sink, but the OCO-2 inversions indicate a decrease.

Over the land, the regional net CO_2_ flux anomalies in Fig. S3 reveal broadly consistent patterns between the DGVMs and the inversions, with both datasets showing widespread transitions from sinks to sources across key regions in 2024, relative to the 2015–2022 average, a reference period which corresponds to the OCO-2 observations. In the Amazon, CO_2_ flux negative anomalies (less uptake or more emissions) reached 1.17 ± 0.15 GtC yr^−1^ in the DGVMs and 0.40 ± 0.26 GtC yr^−1^ in the inversions, reflecting severe drought and fire-related carbon losses during the second part of the year (Fig. S3). In central Africa, the estimated negative anomalies were 0.53 ± 0.42 GtC yr^−1^ (DGVMs) and 0.20 ± 0.14 GtC yr^−1^ (inversions), indicating a marked degradation in the land sink under heat and moisture stress (Fig. S4). In tropical Asia, the DGVMs simulated a smaller negative flux anomaly of 0.15 ± 0.05 GtC yr^−1^, whereas the inversions indicated a weak positive (higher sink) anomaly of 0.10 ± 0.18 GtC yr^−1^. In the northern land areas (north of 30°N), both approaches found the land biosphere to be a net abnormal source of CO_2_, with negative flux anomalies of 0.64 ± 0.91 GtC yr^−1^ (DGVMs) and 0.11 ± 0.26 GtC yr^−1^ (inversions) consistent with a continued weakening of the boreal and temperate land sinks [15].

Over tropical lands, we found a negative CO_2_ flux anomaly, thus a weaker sink or an enhanced source compared to the mean over 2015–2022, persisting from the first to the third quarter of 2024, and easing only in the fourth quarter. During this period, negative flux anomalies persisted over the Amazon even after the El Niño event terminated, while central Africa transitioned into a sink anomaly. These patterns showed fair spatial consistency between the mean flux anomalies from DGVMs and inversions (Fig. S3). Over tropical South America, anomalous CO_2_ sources persisted during JFM-2024, but had a weaker magnitude than in late 2023 (see Fig. 2 of Ref. [15]). However, severe negative CO_2_ flux anomalies re-emerged during JAS-2024, driven primarily by intensified drought conditions and widespread fires. Both the DGVMs and the OCO-2 inversions identify a negative CO_2_ flux anomaly along Brazil’s eastern coast (stronger in the DGVMs). The DGVMs further simulate an additional (weaker) near-neutral to weakly negative anomaly over southern Brazil and a strong negative anomaly in the south-western/central Amazon, whereas the inversions depict these latter regions as weaker and closer to neutral. Considering the absolute values of CO_2_ fluxes instead of anomalies over tropical lands, we estimate that the tropical lands were a net CO_2_ emission of 1.26 GtC yr^−1^ in 2024 compared to 0.22 GtC yr^−1^ in 2023 in the DGVMs, and 0.95 GtC yr^−1^ in 2024 compared to 0.86 GtC yr^−1^ in 2023 in inversions. In total, 52% of tropical land area acted as a net CO_2_ emission to the atmosphere in 2024 in the DGVMs compared to 14% in the inversions. In comparison, during the first part of the El Niño in 2023, 55% and 16% of the tropical lands were net CO_2_ emissions in the DGVMs and inversions, respectively. During the previous extreme El Niño event of 2015–16, 54% and 12% of the tropical lands were net CO_2_ emissions in the DGVMs and inversions, respectively.

Persistent warming and widespread extreme events in 2024 [16] led to a further reduction of the land carbon uptake, with both Northern and tropical regions showing continued and severe sink weakening, extending the trend observed in 2023. Both DGVMs and OCO-2–based inversions indicate that tropical land areas became an even stronger source in 2024, reaching net emissions of 1.26 GtC yr^−1^ and 0.95 GtC yr^−1^, respectively (Fig. S5). In the northern lands (>30°N), the net land carbon sink continued its long-term decline that was identified after 2015, dropping further in 2024 to 0.90 GtC yr^−1^ (0.40 GtC yr^−1^ in DGVMs and 1.41 GtC yr^−1^ in inversions)—well below its initial value of 1.60 GtC yr^−1^ (1.37 GtC yr^−1^ in DGVMs and 1.83 GtC yr^−1^ in inversions) in 2015, and marking a further loss of sink strength following the sharp reduction already observed in 2023. This decline coincides with record-breaking temperatures—2024 being the first year where the global annual mean surface temperature exceeded 1.5°C above pre-industrial levels across all major datasets [16]. These findings reinforce emerging evidence that hot and dry conditions—now becoming increasingly more frequent—can significantly suppress photosynthesis, increase fire activity, and weaken or even reverse land carbon uptake.

For regional fire emissions in 2024, as estimated by the GFAS [12], South America led the surge in wildfire-related carbon emissions. Bolivia registered its highest wildfire carbon emissions since records began, Venezuela reached a new annual record, and Brazil’s fires were exceptional—0.18 GtC yr^−1^ in the Legal Amazon (the highest since 2010) and 0.02 GtC yr^−1^ in Mato Grosso do Sul alone, fueled by severe drought in the Pantanal and eastern Amazon. North America followed, with Canada recording its second-highest annual emissions (behind 2023) and large spring-to-summer events from British Columbia, while western-US fires such as California’s Park Fire pushed national totals near the long-term mean.

Finally, we compared in Fig. S6 the development of flux anomalies for each tropical continent between January of the origin year and December of the peak year for the 2023–24, 2015–16 and 1997–98 El Niños. Although the severity of the 2023–24 event was not as pronounced as the previous ones (Table S1), it is characterized by a larger area of the Amazon and equatorial Africa being under extreme and severe drought according to the ERA5 climate reanalysis, and by larger CO_2_ losses during the origin year and the peak year. Even after the 2023–24 El Niño terminated in April or May 2024, extreme drought persisted in the Amazon during the dry season of 2024 and compounded the two events to cause even larger losses than the dry season of the previous year, with large fire emissions. In tropical Africa, both the first wet season in April 2024 and the second dry season peaking in June 2024 were marked by extreme rainfall deficits according to ERA5, leading to a CO_2_ loss ∼10.7 times larger than the average loss during the same period in the two previous post-El Niño events.

We acknowledge limitations in our approach, including the use of a limited set of DGVMs and inversions than in GCB, although ‘bigger does not mean better’ in terms of model ensemble analysis. Our DGVM simulations are driven by ERA5, which may be drier than alternative forcings (e.g. CRUJRA used in the GCB protocol) and could therefore amplify drought-related reductions in modeled CO_2_ uptake. In addition, some DGVMs may be oversensitive to drought because key hydrological buffering mechanisms, such as groundwater access, deep-water uptake, and lateral redistribution of water to topographically convergent lowlands, are simplified or not represented. Recent evidence suggests for instance that the drought resilience and vulnerability of the Amazon rainforest is strongly modulated by water-table depth and ground-water access, with protective effects that can weaken under prolonged drought, and that lateral hydrologic subsidies can substantially increase effective water availability in lowlands, particularly during dry seasons [17]. We do not know which forcing and hydrological representation is more realistic at large scales, and detailed comparisons should be performed in future work. Finally, land-use change emissions are implicitly included in the DGVM net land flux, but we did not prescribe an explicit land-use change forcing map in our calculations because no low latency map exists that is consistent with historical land use forcing, which is a limitation to our approach.

Taken together, our findings point to a continued decline of the terrestrial biosphere’s capacity to absorb CO_2_, driven by climate extremes such as heatwaves, droughts and fires. This emerging pattern poses a growing risk for positive climate–carbon feedback, in which warming reduces natural carbon uptake, further accelerating atmospheric CO_2_ accumulation. Our low-latency carbon budget framework provides critical early warning signals for the evolving behavior of natural carbon sinks. This approach can inform timely climate policy responses and emphasizes the urgency of mitigating anthropogenic emissions in a rapidly changing Earth system.

## Supplementary Material

nwaf594_Supplemental_File

## Data Availability

The data from Global Carbon Budget 2024 are available at www.icos-cp.eu/science-and-impact/global-carbon-budget/2024. The OCO-2 retrievals are available at disc.gsfc.nasa.gov/datasets?page=1&keywords=OCO-2. NOAA/GML CO_2_ data are available at https://gml.noaa.gov/ccgg/trends/. The Carbon Monitor fossil emissions dataset is available at carbonmonitor.org. The GFED 4.1 s fire emissions dataset is available at geo.vu.nl/∼gwerf/GFED/GFED4/. The GFAS fire emissions dataset is available at atmosphere.copernicus.eu/global-fire-monitoring/. The ERA5 monthly averaged data is available at cds.climate.copernicus.eu/cdsapp#!/dataset/reanalysis-era5-single-levels-monthly-means?tab=overview. The Multivariate ENSO index is available at www.psl.noaa.gov/enso/mei. The GRACE/FO TWS data used in this study are available at www2.csr.utexas.edu/grace/RL06_mascons.html.
