# Supplementary material for: Low latency global carbon budget indicates reduced land carbon sink in the year 2024
Source: Natl Sci Rev. 2026 Jan 15;13(2):nwaf594. doi: 10.1093/nsr/nwaf594 (PMC12860201; doi:10.1093/nsr/nwaf594)
Supplement: nwaf594_Supplemental_File [file nwaf594_supplemental_file.pdf]

Supplemental Information for

Low latency global carbon budget indicates reduced land carbon sink in the year 2024

Philippe Ciais[1,*], Piyu Ke[1,2,3], Yitong Yao[4], Stephen Sitch[3], Wei Li[2], Yidi Xu[1], Xiaomeng Du[2], Xiaofan Gui[5], Ana Bastos[6], Sönke Zaehle[7], Ben Poulter[8], Thomas Colligan[9], Auke M. van der Woude[10], Wouter Peters[10], Zhu Liu[2], Zhe Jin[11], Xiangjun Tian[12], Yilong Wang[12], Junjie Liu[13], Sudhanshu Pandey[13], Chris O'Dell[14], Jiang Bian[5], Chuanlong Zhou[1], John Miller[15], Xin Lan[15,16], Jefferson Goncalves De Souza[3], Michael O'Sullivan[3], Pierre Friedlingstein[3,17], Guido R. van der Werf[10], Glen P. Peters[18], Frédéric Chevallier[1]

1. Laboratoire des Sciences du Climat et de l'Environnement, University Paris Saclay CEA CNRS, Gif sur Yvette 91191, France
2. Department of Earth System Science, Tsinghua University, Beijing 100084, China
3. Faculty of Environment, Science and Economy, University of Exeter, Exeter EX4 4QF, United Kingdom
4. Department of Earth and Environmental Engineering, Columbia University, New York 10027, USA
5. Machine learning group, Microsoft research, Beijing 100080, China
6. Institute for Earth System Science and Remote Sensing, Leipzig University, Leipzig 04103, Germany
7. Department of Biogeochemical Integration, Max Planck Institute for Biogeochemistry, Jena 07745, Germany
8. Spark Climate Solutions, San Francisco, CA, USA
9. Earth System Science Interdisciplinary Center, University of Maryland, College Park, MD 20740, USA
10. Environmental Sciences Group, Dept of Meteorology and Air Quality, Wageningen University, Wageningen 6708 PB, the Netherlands
11. Institute of Carbon Neutrality, Sino-French Institute for Earth System Science, College of Urban and Environmental Sciences, Peking University, Beijing 100871, China
12. State Key Laboratory of Tibetan Plateau Earth System, Environment and Resources (TPESER), Institute of Tibetan Plateau Research, Chinese Academy of Sciences, Beijing 100101, China
13. Jet Propulsion Laboratory, California Institute of Technology, Pasadena 91011, CA, USA

14. Cooperative Institute for Research in the Atmosphere, Colorado State University, Fort Collins, CO 80523, USA
15. National Oceanic and Atmospheric Administration Global Monitoring Laboratory, CO 80303, USA
16. Cooperative Institute for Research in Environmental Sciences, University of Colorado Boulder, CO 80303, USA
17. Laboratoire de Météorologie Dynamique, IPSL, CNRS, ENS, Université PSL, Sorbonne Université, École Polytechnique, Paris 75005, France
18. CICERO Center for International Climate Research, Oslo 0349, Norway

***Corresponding author.** E-mail: philippe.ciais@cea.fr

This PDF file includes:
Supplementary Figures 1 to 6
Supplementary Tables 1

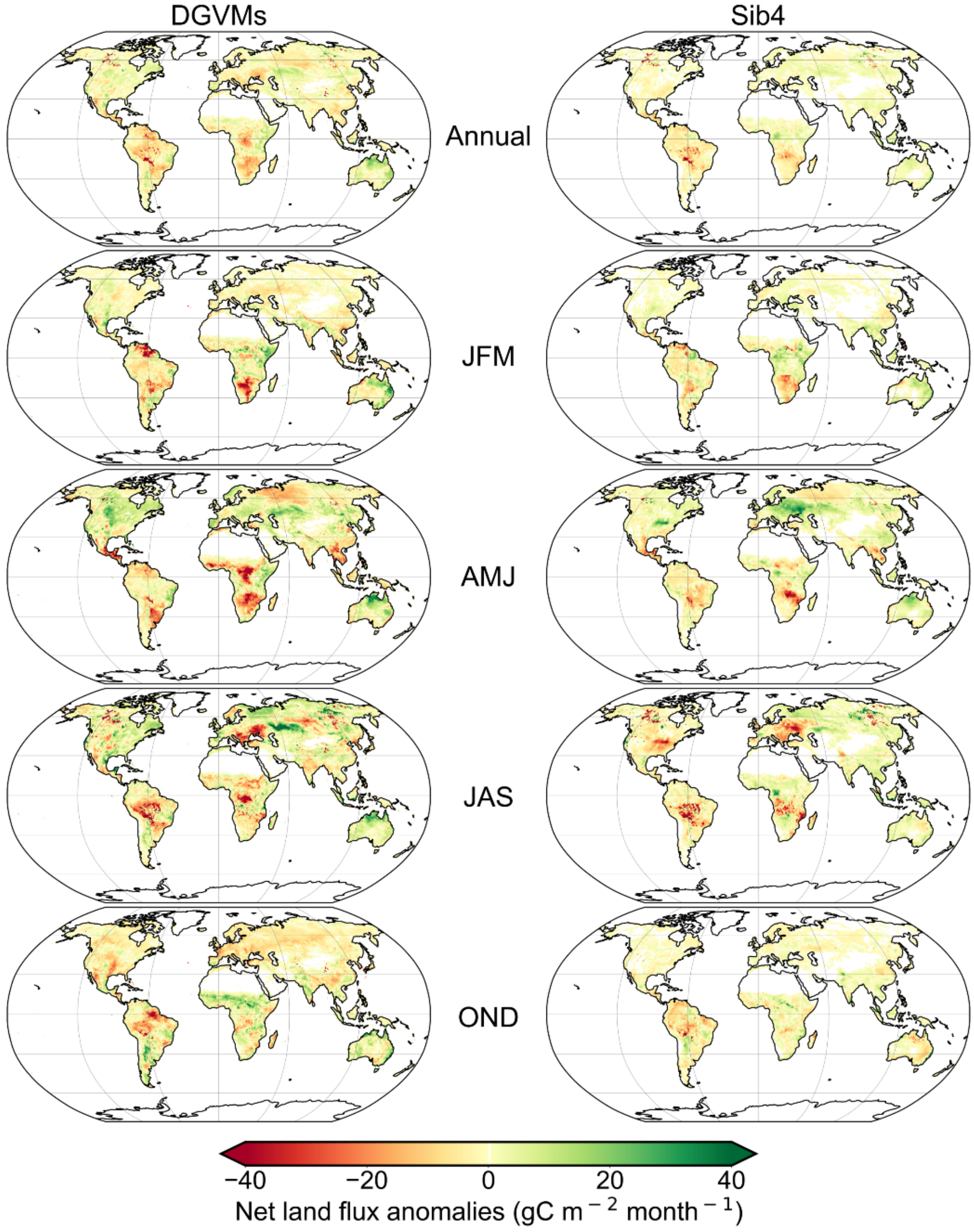

Fig. S1 Net Land CO_2 flux anomalies for each quarter in 2024 compared with the 2015-2022 average for low-latency DGVMs (left column) and the Sib4 model (right column). Positive values represent increased flux from the atmosphere to the land or ocean (carbon sink).

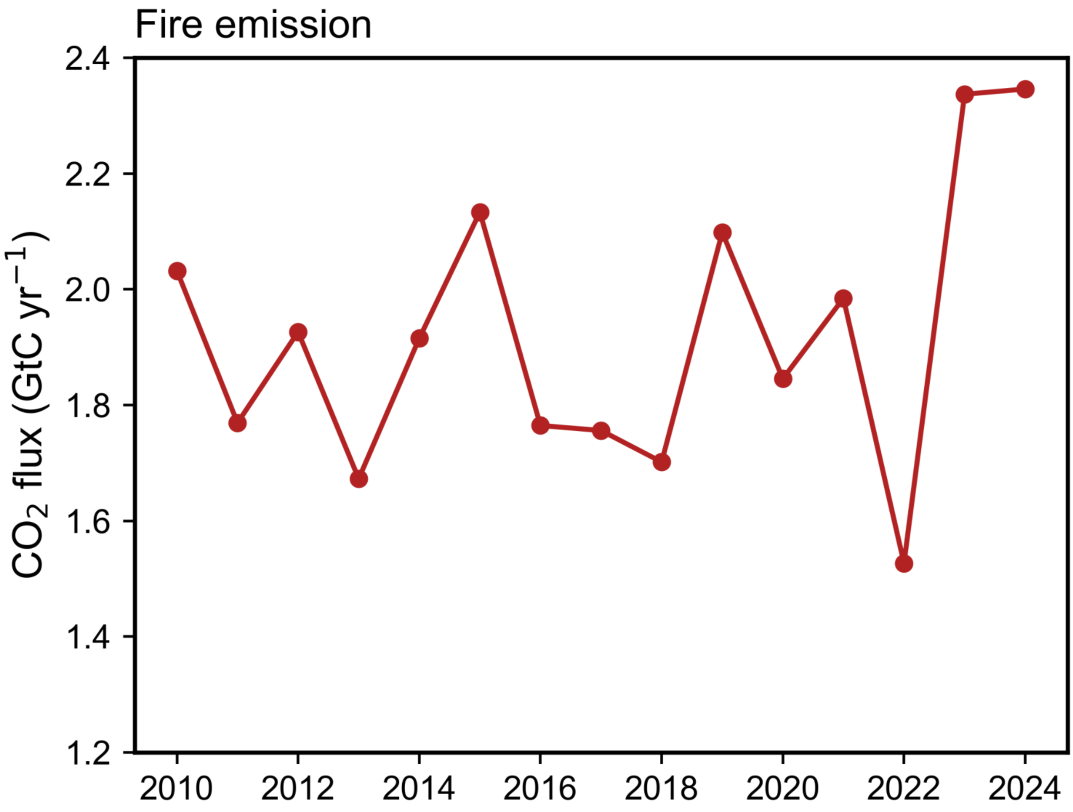

Fig. S2 Wildfire carbon emissions during 2010-2024.

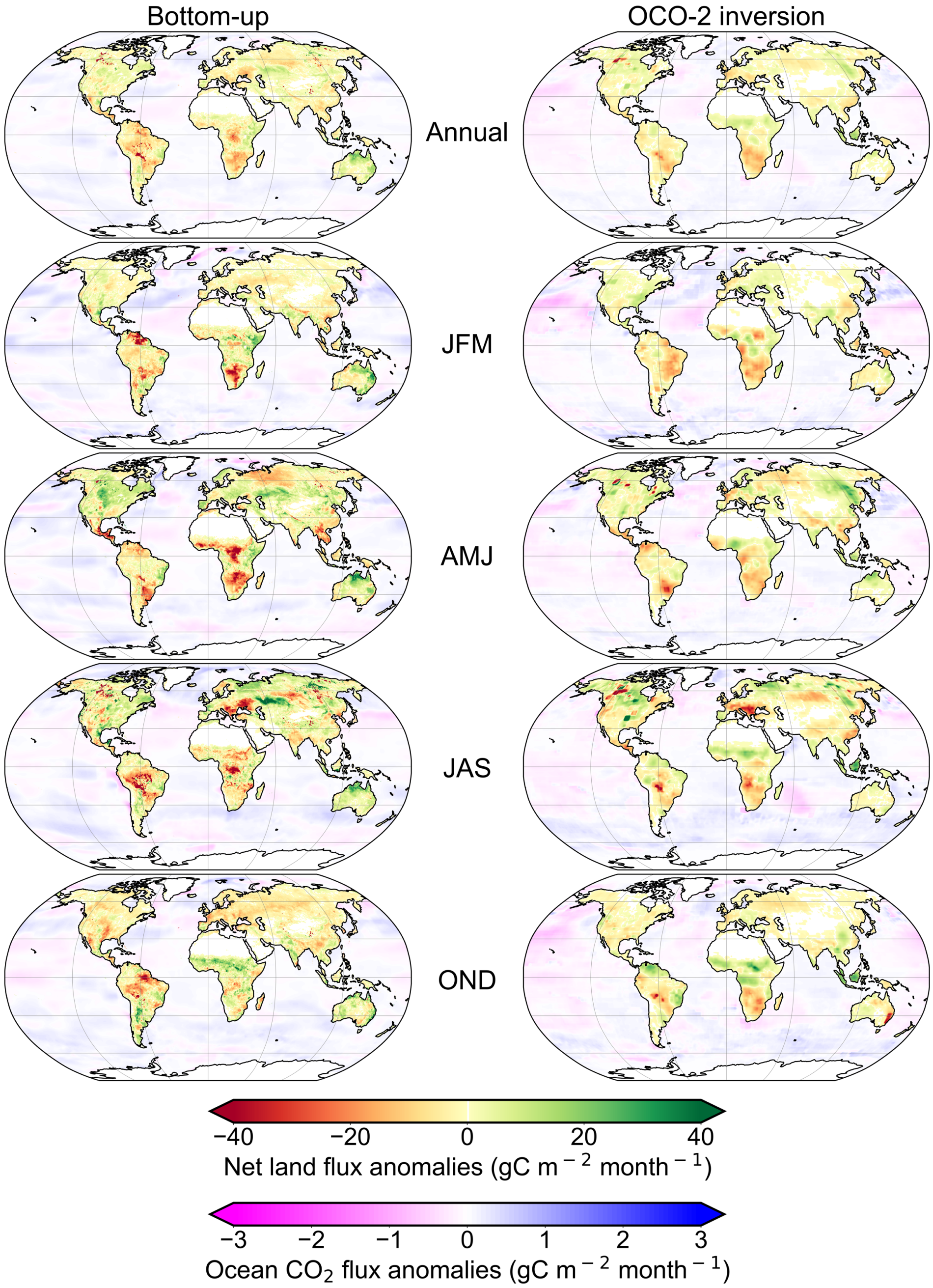

Fig. S3 **Net land and ocean CO_2 flux anomalies for each quarter in 2024 compared with the 2015-2022 average for bottom-up models (left column) and the OCO-2 inversion (right column). Positive values represent increased flux from the atmosphere to the land or ocean (carbon sink).**

a

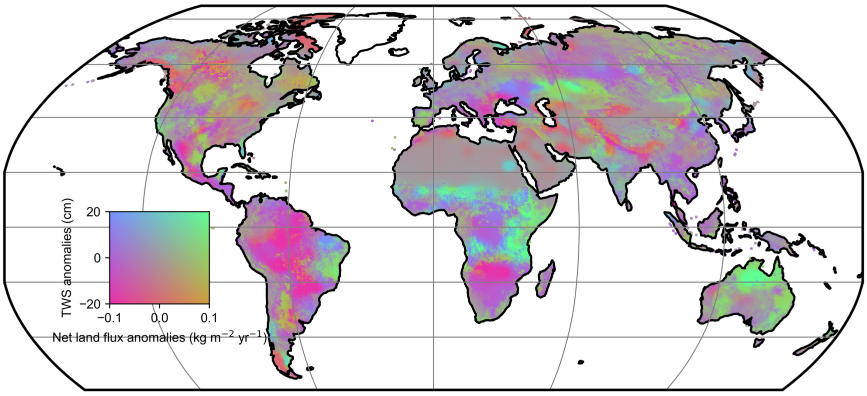

b

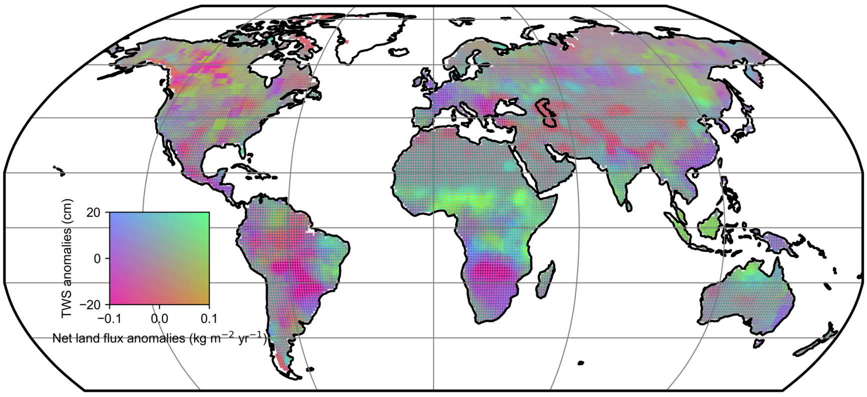

Fig. S4 Bivariate plots showing co-variations between net land flux anomalies from (a) DGVMs and (b) inversions and total water storage anomalies from the GRACE satellites in 2024. Green areas show wetter anomalies coincident with more CO_2 uptake, and magenta areas show dryer anomalies that are coincident with reduced CO_2 uptake.

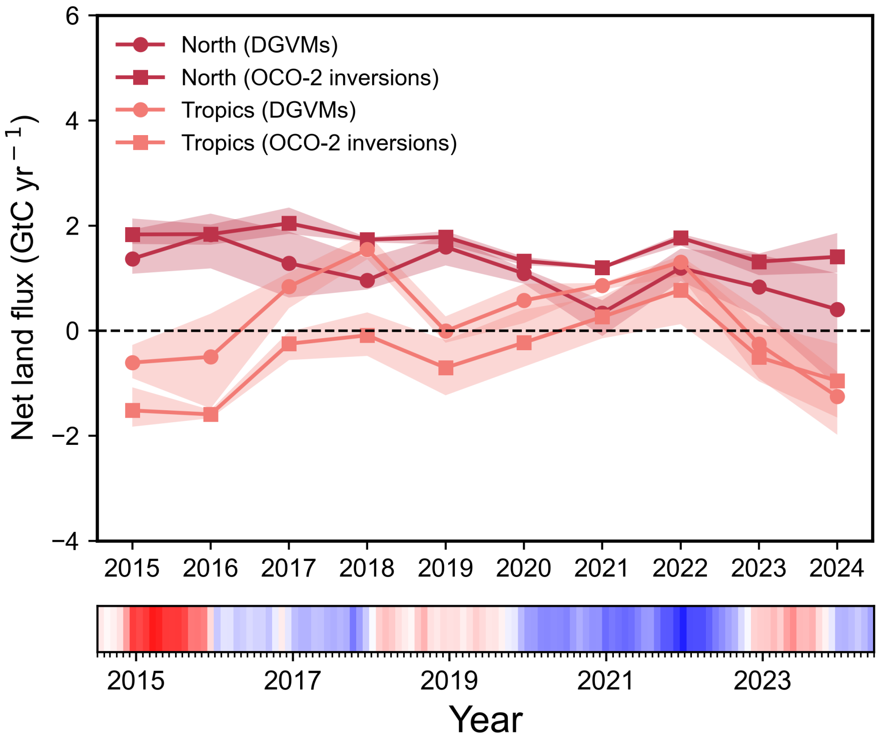

Fig. S5 Changes in the declining northern land sink and the variations of tropical land flux with sources in 2015-16 and 2023-24, from the bottom-up DGVMs and the OCO-2 inversions, during the period from 2015 to 2024. The ENSO index from NOAA Physical Sciences Laboratory (https://www.psl.noaa.gov/enso/mei) is represented in the bottom, with the extreme El Niño of 2015-16, the La Niña from mid 2020 to mid 2023, and the moderate El Niño of the second half of 2023 to early 2024.

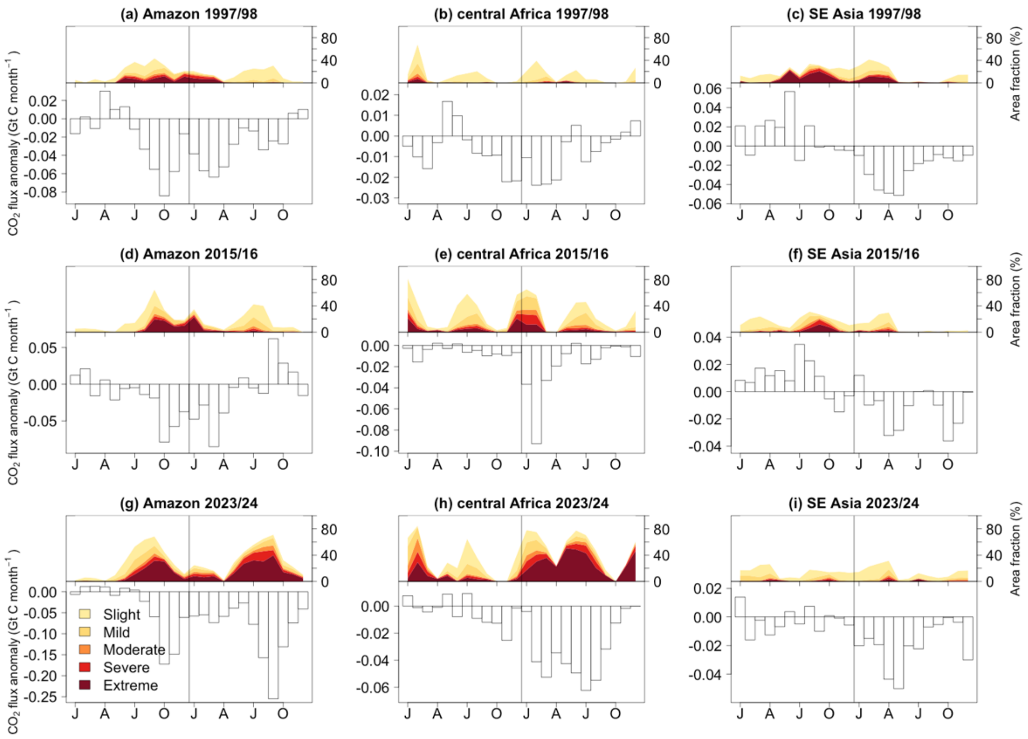

Fig. S6 **Drought timing and extent of the three El Niño events with net CO_2 flux anomalies.** The figure displays monthly net land CO_2 fluxes anomalies from the mean of four DGVMs (ORCHIDEE, OCN, LPJ-EOSIM and JULES) calculated relative to the 5-years pre-event baseline for each El Niño. **(a-c)** For the 1997/98 El Niño in the Amazon, central Africa and tropical Southeast Asia. **(d-f)** Same for the 2015/16 El Niño. **(g-i)** Same for the 2023/24 El Niño.

Table S1 Monthly Multivariate ENSO Index (MEI) values for 1997/98, 2015/16, and 2023/24 El Niño events, obtained from the NOAA Physical Sciences Laboratory (https://www.psl.noaa.gov/enso/mei).

Year	DJ	JF	FM	MA	AM	MJ	JJ	JA	AS	SO	ON	ND
1997	-0.65	-0.72	-0.3	0.15	0.71	2.34	2.26	2.26	2.2	2.06	2.14	2.11
1998	2.3	2.45	2.27	2.61	2.33	0.42	-1.53	-1.82	-1.4	-1.29	-1.34	-1.29
2015	0.23	0.06	0.15	0.31	0.95	1.9	1.79	1.95	2.24	2.15	1.94	1.93
2016	1.94	1.81	1.32	1.33	1.24	0.36	-0.53	-0.27	-0.29	-0.54	-0.48	-0.37
2023	-1.11	-0.91	-0.76	-0.37	-0.06	0.43	0.5	0.51	0.68	0.48	0.91	1.13
2024	0.7	0.68	0.78	0.34	0.12	-0.23	-0.72	-0.73	-0.65	-0.52	-0.68	-0.91
